# Medical Observations and Inquiries

**Published:** 1785

**Authors:** 


VI. *Medical Obſervations and Inquiries. By* A Society of Phyſicians in London. Volume VI. 8vo. Cadell, *London,* 1784. 420 pages.

IN the preface to this volume, which in the value of the materials of which it is compoſed, is in no reſpect inferior to any of the former volumes of this excellent collection, the editors make honourable mention of Dr. Fothergill, Dr. Solander, and Dr. Hunter, three of their aſſociates, lately deceaſed. To theſe irreparable loſſes, which the public, as well as the Society, have to regret, is aſcribed the tardineſs with which the preſent volume makes its appearance. The papers it contains are as follow, viz.

1. *Hiſtory of an Extravaſation of Blood into the Pericardium. By* Jer. Wright, *Surgeon.*—The ſubject of this caſe, which occurred in the year 1749, was a ſtrong and healthy dragoon, twenty-eight years old. He had been employed in lifting ſome heavy cheſts, when he ſuddenly fell down, and complained of a great ſenſe of cold, and of ſomething riſing in his throat that ſeemed to choak him. His countenance appeared livid and bloated, and he was troubled with giddineſs, and frequent inclination to vomit. He was not ſenſible of much pain, neither could he explain the ſeat of his diſorder. Sometimes his reſpiration was interrupted by ſpaſms, and then he ſeemed to be in great agony; but the moſt extraordinary circumſtance of his caſe was, that no pulſation of the heart or arteries was perceptible by the touch. Theſe ſymptoms continued with little variation till his death, which happened ſuddenly about forty-eight hours after the firſt attack, and juſt after he had been walking about his chamber. On diſſection, the pericardium was found diſtended with two quarts of coagulated blood, which had been effuſed from a rupture about an inch long in the vena cava, cloſe by the right auricle. This great ſac filled the whole of the anterior part of the thorax, and compreſſed the lungs into a very ſmall ſpace behind it. The heart appeared ſqueezed and ſhrivelled up to half its natural ſize; and the large veins near it were uncommonly diſtended with blood.

11. *Hiſtory of an Angina Pectoris, ſucceſsfully treated. By Dr.* David Macbride.—With this paper our readers, we may preſume, are already acquainted, as it has been publiſhed by the author author in the fecond edition of his Introduction to the Theory and Practice of Phyfic, and likewife by Dr. Duncan in the 5th volume of his Medical Commentaries.

III. *Two Cafes of Dropfy, fuccefsfully treated by moderate Dofes of Opium. By Mr.* John Mafon, *Surgeon at Leicefter.*—In both thefe cafes, painful fymptoms attended, which indicated the ufe of Opium, and by means of this medicine, given in fmall dofes, the pain was abated, the difcharge of urine was increafed, and the dropfical complaints were foon removed.

IV. *Cafe of an Aneurifm in the Aorta, and in the left carotid Artery, which burft into the Trachea. By Mr.* John Hall, *of Broad Street, Surgeon; late Surgeon to the Leicefter Infirmary.*—The different enlargements of the arteries in this cafe, ferve to confirm the doctrine that aneurifms are often owing to a weaknefs in the arterial fyftem, rather than to local injury. Annexed to the account of the cafe, which appears to be drawn up with great accuracy, is an engraving of the aneurifms as they appeared on diffection.

V. *The Cafe of Mr. Holder. By Mr.* Richard Brown Chefton, *Surgeon at Gloucefter.*— This cafe contains many curious particulars, tending to prove the exiftence of polypous concretions cretions during life; but this account of it, as Mr. Cheſton himſelf has informed us, was written in haſte, and without any expectation of its being publiſhed. He has, therefore, been ſo good as to favour us with a more correct copy of it, with ſome additional remarks, which will be inſerted in the third part of this volume. see P. 225.

VI. *A ſucceſsful Treatment of a ſuppoſed Hydrocephalus internus. By Dr.* Mathew Dobſon.

VII. *A further Account of the ſucceſsful Treatment of a ſuppoſed Hydrocephalus internus. By Dr.* John Hunter.

VIII. *Apparent Effects of Mercury, in Caſes that were ſuppoſed Hydrocephalous. By Dr.* Haygarth.

The caſes which are the ſubject of this and the two preceding articles, all ſerve to confirm the efficacy of mercury in removing ſymptoms ſuppoſed to be occaſioned by dropſy in the brain. The ſubject of Dr. Dobſon's caſe was a child between three and four years old, the youngeſt of four, three of whom had already fallen victims to hydrocephalus internus, as had appeared on diſſection. The ſymptoms in this caſe were a great frequency and irregularity of the pulſe, a dilatation of the pupils, and a great degree of ſtrabiſmus. A mercurial courſe was purſued, till till a moderate ptyalifm was excited, and the fymptoms then began to decline, and in a few days were entirely removed. The ftrabifmus, we are told, was the laft fymptom that difappeared.

In Dr. Hunter's patient, a girl about two years old, the fymptoms of the difeafe feem to have been ftill more decifively marked than in the cafe related by Dr. Dobfon. The child was in a comatofe ftate; the mould of her head, as well as the fize of the head itfelf, was, by her mother's account, fenfibly enlarged fince her difeafe had come on; and the hairy fcalp was covered with many large blue veins, which were greatly diftended on the patient's making any effort in coughing. She often put her hand to her head, and fhrieked out violently from time to time. Her pulfe was quick, and the pupil of her eye, though not much dilated, was totally infenfible to light. Calomel was adminiftered in fmall dofes, and about the ninth day, a confiderable difcharge of faliva came on. So early as the fourth or fifth day of this courfe the fymptoms began to amend, and on the 12th, the patient began to recover her fenfes, but fhe remained blind above a fortnight after her health had been re-eftablifhed in other refpects.

This

This ſymptom gradually went off, and in about ſix weeks her ſight was nearly as good as ever, and the large veins on her head had entirely diſappeared.

In Dr. Haygarth's paper ſeveral caſes are related. The firſt is the caſe of an unmarried lady, twenty-two years old, who had for two months complained of a violent head-ach, which chiefly affected one ſide of her head, and was increaſed by motion, ſound, or light, to an excruciating degree. Her pulſe was remarkably ſlow, often beating only ſixty ſtrokes in a minute, and, when her ſymptoms were violent, not more than fifty-ſeven. She was ſubject to violent retchings and cough. Her urine was turbid, and in ſmall quantity, and ſhe was very thirſty. After other medicines had been tried, without procuring relief, mercurials were adminiſtered cautiouſly till the patient's breath acquired a ſtrong fœtor, and the ſymptoms were then conſiderably relieved, but not removed, till two or three weeks after; ſince which time, we are told, ſhe has enjoyed good health. No ſalivation was produced in this caſe; and we find the author very candidly obſerving on the whole of this hiſtory, that it is by no means calculated to eſtabliſh a new doctrine, trine, neither the difeafe itfelf being fo evident as to be incontrovertible, nor the effects of the remedy clearly afcertained, as fhe ufed various medicines befides mercurials, to relieve her various fymptoms. This cafe is followed by another, which terminated fatally, but in which the relief of fome paralytic fymptoms, under which the patient (a boy fix years old) laboured, is afcribed to the effects of the mercurial courfe that was purfued.

Dr. Haygarth's paper concludes with the cafe of a boy, eight years old. Among other fymptoms, as ftrabifmus, head-ach, urine in fmall quantity, &c. enumerated with great accuracy by the author, we find mention made of coftivenefs, frequent cough, and of the patient's conftantly picking his lips and nofe. Four grains of calomel were adminiftered every day, except one, till he had taken twenty-four grains. Soon after the firft dofe, we are told, he voided a large round worm, and about ten hours after the fecond dofe, he began to recover, and foon got quite well. The calomel produced no other fenfible effect than two or three ftools daily, and an increafed fecretion of urine. Dr. Haygarth is aware, that fome readers may be difpofed to attribute this fever to worms; but if there be

 fuch

ſuch a diſeaſe as a worm fever, (which, he tells us, he has long doubted) he cannot think it was occaſioned by the worm that was voided, becauſe the ſymptoms did not abate for thirty hours. Upon the whole, he thinks it cannot be doubted, that the diſeaſe was a dropſy in the brain. To be able to form an opinion on this point, the reader muſt peruſe the whole of the caſe. For our own parts, when we conſider the ſymptoms of the diſeaſe, and the effect of the medicine, we feel ourſelves diſpoſed to aſcribe the complaint to ſome irritating cauſe in the *primæ viæ*, rather than to any preternatural accumulation of water in the brain.

[ *To be continued.* ]

